# 4-[(*E*)-({4-[Bis­(2-hy­droxy­eth­yl)amino]­phen­yl}imino)­meth­yl]phenol

**DOI:** 10.1107/S1600536810021926

**Published:** 2010-06-16

**Authors:** Xiaoju Liu, Bingqin Yang, Maxim V. Borzov

**Affiliations:** a Key Laboratory of Synthetic and Natural Chemistry of the Ministry of Education, College of Chemistry and Material Science, the North-West University of Xi’an, Taibai Bei Avenue 229, Xi’an 710069, Shaanxi Province, People’s Republic of China

## Abstract

In the title compound, C_17_H_20_N_2_O_3_, the amino N atom is in a planar environment (sum of angles = 360.0°). All hy­droxy H atoms are involved in hydrogen bonding. In the crystal structure, two O—H⋯O and an O—H⋯N_imino_ hydrogen bond result in the formation of a three-dimensional network. The latter hydrogen bonding causes distortion of the planarity of the 4-HO–C_6_H_4_–CH=N–C_6_H_4_– fragment by rotation around the =N—C_Ph_ bond. The crystal studied was a non-merohedral twin [refined BASF parameter for the major component = 0.5293 (7)].

## Related literature

For Schiff bases of the general type *p*-*R*′–C_6_H_4_–CH=N–C_6_H_4_–*R*′′-*p*, see: von König *et al.* (1982 ▶); Haldavanekar *et al.* (2009 ▶); Ferlin *et al.* (2004 ▶); Lewis *et al.* (2009 ▶). For the only two structurally characterized compounds of the type with *R*′′ = *N*(alk­yl)_2_, see: Nagao *et al.* (2002 ▶); Nakai *et al.* (1976 ▶). For the structure of 2,2′-(4-{[(1*E*)-(4-meth­oxy­phen­yl)methyl­ene]amino}­phenyl­imino) bis­ethanol, see: Liu *et al.* (2010 ▶). For the preparation, see: Cho & Park (1997 ▶); Ferlin *et al.* (2004 ▶); von König *et al.* (1982 ▶). For a description of the Cambridge Structural Database, see: Allen (2002 ▶).
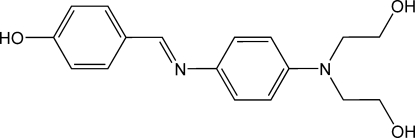


## Experimental

### 

#### Crystal data

C_17_H_20_N_2_O_3_
*M* *_r_* = 300.35
Monoclinic, 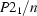

*a* = 10.1426 (9) Å
*b* = 9.5192 (9) Å
*c* = 15.8600 (14) Å
β = 92.679 (1)°
*V* = 1529.6 (2) Å^3^
*Z* = 4
Mo *K*α radiation
μ = 0.09 mm^−1^
*T* = 296 K
0.36 × 0.27 × 0.13 mm

#### Data collection

Bruker SMART APEXII diffractometer
Absorption correction: multi-scan (*TWINABS*; Sheldrick, 1996 ▶) *T*_min_ = 0.968, *T*_max_ = 0.988
5506 measured reflections
5506 independent reflections
2959 reflections with *I* > 2σ(*I*)

#### Refinement

*R*[*F* ^2^ > 2σ(*F* ^2^)] = 0.047
*wR*(*F* ^2^) = 0.109
*S* = 0.84
5506 reflections
217 parameters
H atoms treated by a mixture of independent and constrained refinement
Δρ_max_ = 0.16 e Å^−3^
Δρ_min_ = −0.18 e Å^−3^

### 

Data collection: *APEX2* (Bruker, 2007 ▶); cell refinement: *SAINT* (Bruker, 2007 ▶); data reduction: *SAINT*; program(s) used to solve structure: *SHELXS97* (Sheldrick, 2008 ▶); program(s) used to refine structure: *SHELXL97* (Sheldrick, 2008 ▶); molecular graphics: *SHELXTL* (Sheldrick, 2008 ▶) and *OLEX2* (Dolomanov *et al.*, 2009 ▶); software used to prepare material for publication: *SHELXL97* and *OLEX2*.

## Supplementary Material

Crystal structure: contains datablocks I, global. DOI: 10.1107/S1600536810021926/hg2687sup1.cif

Structure factors: contains datablocks I. DOI: 10.1107/S1600536810021926/hg2687Isup2.hkl

Additional supplementary materials:  crystallographic information; 3D view; checkCIF report

## Figures and Tables

**Table 1:** Hydrogen-bond geometry (Å, °)

*D*—H⋯*A*	*D*—H	H⋯*A*	*D*⋯*A*	*D*—H⋯*A*
O1—H1⋯O3^i^	0.89 (2)	1.82 (2)	2.669 (2)	160 (2)
O2—H2⋯N1^ii^	0.95 (2)	1.82 (2)	2.771 (2)	172 (2)
O3—H3⋯O2^iii^	0.89 (2)	1.79 (2)	2.674 (2)	174 (2)

Symmetry codes: (i) 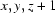; (ii) 
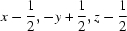
; (iii) 
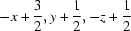
.

**Fig. 1.**
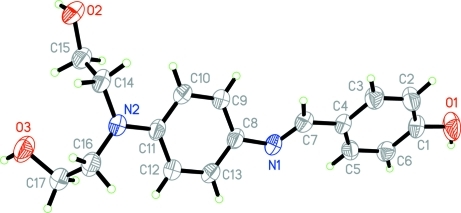
Asymmetric unit of the compound (I) with labelling and thermal ellipsoids at the 50% probability level.

**Fig. 2.**
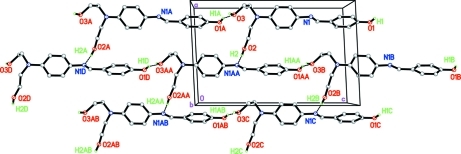
Assembling of the molecules of (I) into a "foldered" zigzag layer. Hydrogen atoms except of the OH ones are omitted for clarity. Labelling is provided only for atoms involved in H-bonding. H-bonds are depicted as dashed lines.

**Fig. 3.**
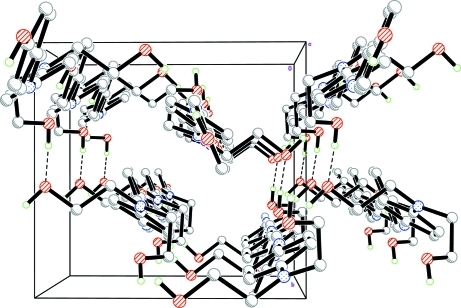
Inter-layer assembling of the molecules of (I) into a 3D-network. Hydrogen atoms except of the OH ones are omitted for clarity. Only O3—H3···O2iii [symmetry code: (iii) –x + 1.5, y + 0.5, –z + 0.5] bonds and their symmetry equivalents are depicted (dashed lines).

## supplementary crystallographic information

### Comment

Schiff bases of general type *p*-R'–C_6_H_4_–CH=N–C_6_H_4_–R"-*p* are well-known objects that find their practical application in various areas [photography (for instance, see von König *et al.*, 1982), medicinal and pharmaceutical chemistry (for instance, see Haldavanekar *et al.*, 2009; Ferlin *et al.*, 2004; Lewis *et al.*, 2009)]. Recently we were interested in preparation of a series of 2,2'-(4-{[(1*E*)-phenylmethylene]amino}phenylimino)bisethanols as semi-products for their further conversion into paracyclophanes. This way, 4-[(*E*)-({4-[bis(2-hydroxyethyl)amino]phenyl}imino)methyl]phenol, C_17_H_20_N_2_O_3_, (I), and 2,2'-(4-{[(1*E*)-(4-methoxyphenyl)methylene]amino}phenylimino)bisethanol, C_18_H_22_N_2_O_3_, [II; Liu *et al.* (2010)], were prepared by a condensation reaction between 2,2'-[(4-aminophenyl)imino]bisethanol and 4-methoxy- or 4-hydroxybenzaldehyde, respectively (see Scheme).

Despite of the fact that structurally characterized Schiff bases of general type *p*-R'–C_6_H_4_–CH=N–C_6_H_4_–R"-*p* are well presented in the Cambridge Structural Database [CSD; Version 5.27, release February 2009; Allen, 2002; 128 entries, 173 fragments], among them there are only two compounds with R" = *N*(alkyl)_2_ [namely: R' = H, R'' = NEt_2_ (Nagao *et al.*, 2002) and R' = NO_2_, R" = NMe_2_ (Nakai *et al.*, 1976)]. From this viewpoint, X-ray single crystal study of (I) presents a certain descriptive interest.

The asymmetric unit of (I) is shown in Fig. 1. Except of dihedral angle C7–N1–C8–C9, asymmetric units of (I) and [II; Liu *et al.* (2010)] have nearly identical geometries (Supplementary materials). Bond lengths, valency angles and C4–C7–N1–C8 torsion angle values for C4/C7/N1/C8 fragment match well the reported median values for *p*-R'–C_6_H_4_–CH=N–C_6_H_4_–R"-*p* [analysis of the Cambridge Structural Database (CSD); Version 5.27, release February 2009; Allen, 2002; 128 entries, 173 fragments]. Fragments O1/C1—C7/N1 and C8—C13/N2/C14/C16 are nearly planar [within 0.04 and 0.07 Å for (I)]. Atom N2 is also in a planar environment [sum of the valent angles 360.0 (6)°] what presents the most frequent case for aryldialkylamines (range from 317.6 to 360.0°, median value 359.0°)..

All hydroxy H-atoms in (I) are involved into hydrogen bonding [for the H-bonds lengths and angles values see the Table 1]. This way, O1—H1···O3 H-bonds assemble the molecules in chains stretched along the *c*-axis of the crystal lattice [linked molecules are connected by a simple (0,0,±1) translation]. These chains, in their turn, assemble into "foldered" zigzag layers parallel to the *a*0*b* face due to O2—H2···N1 bonds (see Fig. 2). Finally, O3—H3···O2 bonds join the adjacent layers what completes the entire 3D-framework (see Fig. 3). Involving of the N1 imino-atom into H-binding, evidently, causes a considerable distortion of the 4-HO–C_6_H_4_–CH=N–C_6_H_4_– fragment planarity by rotation around the ═N—C_Ph_ bond.

### Experimental

1-Chloro-4-nitrobenzene, 4-methoxy- and 4-hydroxybenzaldehydes, 2,2'-iminobisethanol, ammonium formate, 10% Pd/C catalyst and solvents were purchased from Sinopharm Chemical Reagent and Tianjin Fuyu Chemical companies. 2,2'-[(4-nitrophenyl)imino]bisethanol was prepared as described by Cho & Park (1997) and Ferlin *et al.* (2004). Reduction of the nitro-group was carried out as described by Lewis *et al.* (2009). Schiff-base preparation was done closely to what described by von König *et al.* (1982). – ^1^H NMR spectra were recorded on a Varian INOVA-400 instrument in CD_3_OD at 298 K using the resonance of the residual solvent protons as an internal reference [δ(H) = 3.30 ppm]. Procedure: 1-chloro-4-nitrobenzene (15.76 g, 0.10 mol) was dissolved in 2,2'-iminobisethanol (50 ml). The reaction mixture was heated at 393 K for 10 h, cooled down to room temperature, the precipitated crude 2,2'-[(4-nitrophenyl)imino]bisethanol filtered off, dried in vacuum and recrystallized from minimal amount of hot ethanol. Yield 11.54 g (51%). 2,2'-[(4-nitrophenyl)imino]bisethanol (8.15 g, 0.036 mol) was dissolved in MeOH (50 ml). To this solution, HCOONH_4 _(0.216 mol) and 10% Pd/C (0.6 g) were added and the slurry was stirred at 293 K for 30 min. On removal of the catalyst by filtration, the filtrate was placed into a N_2_-flushed flask containing 1 ml of acetic acid and an equimolar (0.036 mol) amount of 4-hydroxybenzaldehyde (0.036 mol) was added dropwise at 333 K during 30 min. The reaction mixture was kept at the same temperature for additional 30 min, cooled down to 273 K and ice-cold water (200 ml) was added. The precipitated light-green (I) solid was collected by filtration, washed with water, dried under reduced pressure and, finally, re-crystallized by a slow evaporation of their methanol solutions in air at 293 K. Yield 92%, m.p. 476 K. ^1^H NMR for (I) δ: 8.45 (s, 1H, CH═N), 6.78–7.73 (m, 8H, C_6_H_4_), 3.30, 3.74 (both t, 4 H and 4 H, ^3^*J*_HH_ = 7.2 Hz, CH_2_). Single crystal of (I) suitable for X-ray diffraction analysis was picked up directly from the obtained materials.

### Refinement

All non-H atoms were refined anisotropically. H atoms except of H7 and OH group ones were treated as riding atoms with distances C—H = 0.97 (CH_2_), 0.93 Å (C_Ar_H), and *U*_iso_(H) = 1.2*U*_eq_(C). Atoms H7 and OH group H atoms were found from difference Fourier syntheses and refined isotropically.

### Crystal data

**Table:**

C_17_H_20_N_2_O_3_	*F*(000) = 640
*M**_r_* = 300.35	*D*_x_ = 1.304 Mg m^−^^3^
Monoclinic, *P*2_1_/*n*	Mo *K*α radiation, λ = 0.71073 Å
Hall symbol: -P 2yn	Cell parameters from 4620 reflections
*a* = 10.1426 (9) Å	θ = 2.3–27.0°
*b* = 9.5192 (9) Å	µ = 0.09 mm^−^^1^
*c* = 15.8600 (14) Å	*T* = 296 K
β = 92.679 (1)°	Block, green
*V* = 1529.6 (2) Å^3^	0.36 × 0.27 × 0.13 mm
*Z* = 4	

### Data collection

**Table:**

Bruker SMART APEXII diffractometer	5506 measured reflections
Radiation source: fine-focus sealed tube	5506 independent reflections
graphite	2959 reflections with *I* > 2σ(*I*)
Detector resolution: 8.333 pixels mm^-1^	θ_max_ = 25.1°, θ_min_ = 2.3°
phi and ω scans	*h* = −12→12
Absorption correction: multi-scan (*TWINABS*; Sheldrick, 1996)	*k* = 0→11
*T*_min_ = 0.968, *T*_max_ = 0.988	*l* = 0→18

### Refinement

**Table:**

Refinement on *F*^2^	Secondary atom site location: difference Fourier map
Least-squares matrix: full	Hydrogen site location: inferred from neighbouring sites
*R*[*F*^2^ > 2σ(*F*^2^)] = 0.047	H atoms treated by a mixture of independent and constrained refinement
*wR*(*F*^2^) = 0.109	*w* = 1/[σ^2^(*F*_o_^2^) + (0.0702*P*)^2^] where *P* = (*F*_o_^2^ + 2*F*_c_^2^)/3
*S* = 0.84	(Δ/σ)_max_ < 0.001
5506 reflections	Δρ_max_ = 0.16 e Å^−^^3^
217 parameters	Δρ_min_ = −0.18 e Å^−^^3^
0 restraints	Extinction correction: *SHELXL97* (Sheldrick, 2008), Fc^*^=kFc[1+0.001xFc^2^λ^3^/sin(2θ)]^-1/4^
Primary atom site location: structure-invariant direct methods	Extinction coefficient: 0.0107 (15)

### Special details

**Table:**

Experimental. Sample of (I) was a two-component non-merohedral twin with approximately equal component contribution. Thus, the structure of (I) was solved and pre-refined for one of the components (HKLF 4 format) and finally refined for the full set of reflexions (HKLF 5 file format). The refined BASF parameter for the prevailing component equals 0.5293 (7).
Geometry. All esds (except the esd in the dihedral angle between two l.s. planes) are estimated using the full covariance matrix. The cell esds are taken into account individually in the estimation of esds in distances, angles and torsion angles; correlations between esds in cell parameters are only used when they are defined by crystal symmetry. An approximate (isotropic) treatment of cell esds is used for estimating esds involving l.s. planes.
Refinement. Refinement of *F*^2^ against ALL reflections. The weighted *R*-factor wR and goodness of fit *S* are based on *F*^2^, conventional *R*-factors *R* are based on *F*, with *F* set to zero for negative *F*^2^. The threshold expression of *F*^2^ > 2σ(*F*^2^) is used only for calculating *R*-factors(gt) etc. and is not relevant to the choice of reflections for refinement. *R*-factors based on *F*^2^ are statistically about twice as large as those based on *F*, and *R*- factors based on ALL data will be even larger.

### Fractional atomic coordinates and isotropic or equivalent isotropic displacement parameters (Å^2^)

**Table:**

	*x*	*y*	*z*	*U*_iso_*/*U*_eq_	
O1	0.84481 (15)	0.10572 (15)	1.16972 (7)	0.0735 (5)	
O2	0.56639 (11)	0.06213 (12)	0.32409 (7)	0.0477 (3)	
O3	0.90811 (13)	0.32742 (15)	0.26638 (8)	0.0649 (4)	
N1	0.89456 (12)	0.22592 (15)	0.77116 (8)	0.0438 (4)	
N2	0.89798 (12)	0.13765 (13)	0.41928 (7)	0.0423 (4)	
C1	0.84745 (17)	0.11819 (19)	1.08463 (10)	0.0479 (5)	
C2	0.80262 (17)	0.00647 (18)	1.03658 (10)	0.0551 (5)	
H2A	0.7703	−0.0730	1.0628	0.066*	
C3	0.80526 (16)	0.01135 (18)	0.94979 (10)	0.0505 (5)	
H3A	0.7768	−0.0661	0.9181	0.061*	
C4	0.84966 (15)	0.12981 (17)	0.90899 (9)	0.0394 (4)	
C5	0.89081 (15)	0.24390 (17)	0.95828 (9)	0.0434 (4)	
H5	0.9183	0.3256	0.9322	0.052*	
C6	0.89162 (15)	0.23798 (17)	1.04515 (9)	0.0443 (4)	
H6	0.9218	0.3143	1.0772	0.053*	
C7	0.85321 (16)	0.1266 (2)	0.81716 (10)	0.0442 (4)	
C8	0.88878 (15)	0.20652 (16)	0.68171 (9)	0.0401 (4)	
C9	0.78712 (16)	0.13679 (17)	0.63799 (9)	0.0444 (4)	
H9	0.7167	0.1025	0.6673	0.053*	
C10	0.78819 (16)	0.11715 (17)	0.55179 (9)	0.0452 (4)	
H10	0.7175	0.0715	0.5242	0.054*	
C11	0.89302 (15)	0.16422 (16)	0.50488 (9)	0.0385 (4)	
C12	0.99168 (16)	0.24157 (17)	0.54925 (9)	0.0449 (4)	
H12	1.0603	0.2802	0.5201	0.054*	
C13	0.98867 (16)	0.26125 (17)	0.63517 (9)	0.0450 (4)	
H13	1.0557	0.3128	0.6627	0.054*	
C14	0.79307 (15)	0.05868 (17)	0.37522 (9)	0.0433 (4)	
H14B	0.7674	−0.0192	0.4103	0.052*	
H14A	0.8258	0.0202	0.3236	0.052*	
C15	0.67400 (15)	0.14873 (17)	0.35403 (10)	0.0442 (4)	
H15B	0.6494	0.1998	0.4038	0.053*	
H15A	0.6948	0.2166	0.3110	0.053*	
C16	1.01067 (15)	0.17981 (18)	0.37224 (10)	0.0479 (5)	
H16A	1.0211	0.1130	0.3269	0.057*	
H16B	1.0892	0.1743	0.4094	0.057*	
C17	1.00283 (17)	0.32486 (19)	0.33494 (10)	0.0536 (5)	
H17A	0.9782	0.3916	0.3777	0.064*	
H17B	1.0885	0.3519	0.3155	0.064*	
H1	0.880 (2)	0.183 (2)	1.1930 (13)	0.107 (9)*	
H2	0.5011 (18)	0.130 (2)	0.3071 (11)	0.086 (7)*	
H3	0.913 (2)	0.408 (2)	0.2387 (13)	0.117 (9)*	
H7	0.8211 (14)	0.0378 (16)	0.7921 (9)	0.050 (5)*	

### Atomic displacement parameters (Å^2^)

**Table:**

	*U*^11^	*U*^22^	*U*^33^	*U*^12^	*U*^13^	*U*^23^
O1	0.1199 (13)	0.0705 (11)	0.0296 (8)	−0.0099 (9)	−0.0027 (7)	0.0057 (7)
O2	0.0524 (8)	0.0448 (8)	0.0442 (7)	0.0026 (7)	−0.0141 (6)	−0.0081 (6)
O3	0.0834 (10)	0.0586 (9)	0.0499 (8)	−0.0142 (8)	−0.0269 (7)	0.0155 (7)
N1	0.0480 (9)	0.0525 (10)	0.0303 (8)	−0.0016 (7)	−0.0057 (6)	0.0003 (7)
N2	0.0449 (8)	0.0523 (9)	0.0290 (8)	−0.0043 (7)	−0.0030 (7)	0.0018 (6)
C1	0.0604 (12)	0.0515 (12)	0.0314 (10)	0.0057 (10)	−0.0029 (9)	0.0022 (8)
C2	0.0775 (13)	0.0447 (12)	0.0430 (11)	−0.0039 (10)	0.0006 (10)	0.0081 (9)
C3	0.0674 (13)	0.0429 (11)	0.0406 (11)	−0.0041 (9)	−0.0050 (9)	−0.0011 (8)
C4	0.0432 (10)	0.0426 (10)	0.0319 (9)	0.0031 (8)	−0.0042 (8)	0.0015 (8)
C5	0.0492 (11)	0.0445 (11)	0.0365 (10)	−0.0023 (9)	0.0010 (8)	0.0039 (8)
C6	0.0508 (11)	0.0455 (11)	0.0364 (10)	−0.0008 (9)	−0.0017 (8)	−0.0056 (8)
C7	0.0466 (11)	0.0480 (12)	0.0373 (11)	0.0008 (9)	−0.0057 (8)	−0.0017 (9)
C8	0.0452 (10)	0.0437 (10)	0.0307 (9)	0.0000 (8)	−0.0061 (8)	0.0004 (8)
C9	0.0448 (10)	0.0548 (11)	0.0333 (10)	−0.0069 (9)	−0.0025 (8)	0.0042 (8)
C10	0.0473 (10)	0.0521 (11)	0.0352 (10)	−0.0096 (9)	−0.0087 (8)	0.0014 (8)
C11	0.0440 (10)	0.0404 (10)	0.0303 (9)	0.0020 (8)	−0.0073 (8)	0.0050 (7)
C12	0.0445 (10)	0.0555 (12)	0.0343 (10)	−0.0052 (9)	−0.0036 (8)	0.0060 (8)
C13	0.0456 (10)	0.0515 (12)	0.0370 (10)	−0.0068 (9)	−0.0092 (8)	0.0010 (8)
C14	0.0521 (10)	0.0451 (11)	0.0318 (9)	0.0012 (9)	−0.0067 (8)	−0.0011 (8)
C15	0.0486 (10)	0.0451 (11)	0.0383 (10)	−0.0015 (9)	−0.0066 (8)	−0.0017 (8)
C16	0.0472 (10)	0.0608 (12)	0.0351 (10)	0.0070 (9)	−0.0035 (8)	0.0014 (8)
C17	0.0553 (11)	0.0654 (13)	0.0392 (10)	−0.0113 (10)	−0.0088 (9)	0.0078 (9)

### Geometric parameters (Å, °)

**Table:**

O1—C1	1.3563 (18)	C7—H7	0.984 (15)
O1—H1	0.89 (2)	C8—C13	1.383 (2)
O2—C15	1.4310 (17)	C8—C9	1.385 (2)
O2—H2	0.952 (19)	C9—C10	1.3805 (19)
O3—C17	1.4172 (18)	C9—H9	0.9300
O3—H3	0.89 (2)	C10—C11	1.400 (2)
N1—C7	1.277 (2)	C10—H10	0.9300
N1—C8	1.4292 (18)	C11—C12	1.405 (2)
N2—C11	1.3843 (18)	C12—C13	1.3773 (19)
N2—C16	1.4504 (18)	C12—H12	0.9300
N2—C14	1.4546 (18)	C13—H13	0.9300
C1—C2	1.373 (2)	C14—C15	1.506 (2)
C1—C6	1.385 (2)	C14—H14B	0.9700
C2—C3	1.379 (2)	C14—H14A	0.9700
C2—H2A	0.9300	C15—H15B	0.9700
C3—C4	1.386 (2)	C15—H15A	0.9700
C3—H3A	0.9300	C16—C17	1.503 (2)
C4—C5	1.391 (2)	C16—H16A	0.9700
C4—C7	1.459 (2)	C16—H16B	0.9700
C5—C6	1.3786 (19)	C17—H17A	0.9700
C5—H5	0.9300	C17—H17B	0.9700
C6—H6	0.9300		
			
C1—O1—H1	108.4 (14)	C9—C10—H10	119.2
C15—O2—H2	102.4 (11)	C11—C10—H10	119.2
C17—O3—H3	110.1 (14)	N2—C11—C10	121.75 (14)
C7—N1—C8	118.17 (14)	N2—C11—C12	121.97 (14)
C11—N2—C16	121.30 (13)	C10—C11—C12	116.28 (14)
C11—N2—C14	120.41 (13)	C13—C12—C11	121.25 (15)
C16—N2—C14	118.18 (12)	C13—C12—H12	119.4
O1—C1—C2	117.58 (16)	C11—C12—H12	119.4
O1—C1—C6	122.93 (16)	C12—C13—C8	121.84 (15)
C2—C1—C6	119.49 (15)	C12—C13—H13	119.1
C1—C2—C3	120.43 (16)	C8—C13—H13	119.1
C1—C2—H2A	119.8	N2—C14—C15	111.96 (13)
C3—C2—H2A	119.8	N2—C14—H14B	109.2
C2—C3—C4	121.04 (16)	C15—C14—H14B	109.2
C2—C3—H3A	119.5	N2—C14—H14A	109.2
C4—C3—H3A	119.5	C15—C14—H14A	109.2
C3—C4—C5	117.93 (14)	H14B—C14—H14A	107.9
C3—C4—C7	118.23 (16)	O2—C15—C14	109.72 (13)
C5—C4—C7	123.83 (15)	O2—C15—H15B	109.7
C6—C5—C4	121.16 (15)	C14—C15—H15B	109.7
C6—C5—H5	119.4	O2—C15—H15A	109.7
C4—C5—H5	119.4	C14—C15—H15A	109.7
C5—C6—C1	119.89 (15)	H15B—C15—H15A	108.2
C5—C6—H6	120.1	N2—C16—C17	115.35 (14)
C1—C6—H6	120.1	N2—C16—H16A	108.4
N1—C7—C4	125.38 (17)	C17—C16—H16A	108.4
N1—C7—H7	121.1 (8)	N2—C16—H16B	108.4
C4—C7—H7	113.5 (8)	C17—C16—H16B	108.4
C13—C8—C9	117.40 (14)	H16A—C16—H16B	107.5
C13—C8—N1	118.95 (14)	O3—C17—C16	109.84 (14)
C9—C8—N1	123.64 (14)	O3—C17—H17A	109.7
C10—C9—C8	121.38 (15)	C16—C17—H17A	109.7
C10—C9—H9	119.3	O3—C17—H17B	109.7
C8—C9—H9	119.3	C16—C17—H17B	109.7
C9—C10—C11	121.62 (15)	H17A—C17—H17B	108.2
			
O1—C1—C2—C3	−178.64 (16)	C16—N2—C11—C10	−176.39 (14)
C6—C1—C2—C3	2.0 (3)	C14—N2—C11—C10	−0.2 (2)
C1—C2—C3—C4	−1.7 (3)	C16—N2—C11—C12	4.6 (2)
C2—C3—C4—C5	−0.3 (2)	C14—N2—C11—C12	−179.19 (14)
C2—C3—C4—C7	178.38 (16)	C9—C10—C11—N2	176.24 (14)
C3—C4—C5—C6	2.1 (2)	C9—C10—C11—C12	−4.7 (2)
C7—C4—C5—C6	−176.54 (15)	N2—C11—C12—C13	−176.78 (14)
C4—C5—C6—C1	−1.8 (2)	C10—C11—C12—C13	4.1 (2)
O1—C1—C6—C5	−179.58 (16)	C11—C12—C13—C8	−0.2 (2)
C2—C1—C6—C5	−0.3 (2)	C9—C8—C13—C12	−3.3 (2)
C8—N1—C7—C4	−178.79 (15)	N1—C8—C13—C12	177.43 (14)
C3—C4—C7—N1	−178.17 (16)	C11—N2—C14—C15	79.93 (17)
C5—C4—C7—N1	0.5 (3)	C16—N2—C14—C15	−103.73 (15)
C7—N1—C8—C13	−143.95 (16)	N2—C14—C15—O2	−170.21 (11)
C7—N1—C8—C9	36.9 (2)	C11—N2—C16—C17	−89.41 (17)
C13—C8—C9—C10	2.8 (2)	C14—N2—C16—C17	94.28 (17)
N1—C8—C9—C10	−178.01 (14)	N2—C16—C17—O3	−71.61 (18)
C8—C9—C10—C11	1.3 (2)		

### Hydrogen-bond geometry (Å, °)

**Table:**

*D*—H···*A*	*D*—H	H···*A*	*D*···*A*	*D*—H···*A*
O1—H1···O3^i^	0.89 (2)	1.82 (2)	2.669 (2)	160 (2)
O2—H2···N1^ii^	0.95 (2)	1.82 (2)	2.771 (2)	172 (2)
O3—H3···O2^iii^	0.89 (2)	1.79 (2)	2.674 (2)	174 (2)

Symmetry codes: (i) *x*, *y*, *z*+1; (ii) *x*−1/2, −*y*+1/2, *z*−1/2; (iii) −*x*+3/2, *y*+1/2, −*z*+1/2.
